# Physical activity and sedentary behaviours among rural adults in suixi, china: a cross-sectional study

**DOI:** 10.1186/1479-5868-8-37

**Published:** 2011-04-26

**Authors:** Ding Ding, James F Sallis, Melbourne F Hovell, Jianzhong Du, Miao Zheng, Haiying He, Neville Owen

**Affiliations:** 1 Graduate School of Public Health, San Diego State University, San Diego, California, USA; 2 Department of Family and Preventive Medicine, University of California-San Diego, La Jolla, California, USA; 3 Department of Psychology, San Diego State University, San Diego, California, USA; 4 Department of Chemistry, Zhanjiang Normal University, Zhanjiang, Guangdong, China; 5 Cancer Prevention Research Centre, School of Population Health, University of Queensland, Herston, Queensland, Australia

## Abstract

**Background:**

Modernisation and urbanisation have led to lifestyle changes and increasing risks for chronic diseases in China. Physical activity and sedentary behaviours among rural populations need to be better understood, as the rural areas are undergoing rapid transitions. This study assessed levels of physical activity and sedentary behaviours of farming and non-farming adults in rural Suixi, described activity differences between farming and non-farming seasons, and examined correlates of leisure-time physical activity (LTPA) and TV viewing.

**Methods:**

A random sample of rural adults (n = 287) in Suixi County, Guangdong, China were surveyed in 2009 by trained interviewers. Questionnaires assessed multiple physical activities and sedentary behaviours, and their correlates. Analysis of covariance compared activity patterns across occupations, and multiple logistic regressions assessed correlates of LTPA and TV viewing. Quantitative data analyses were followed by community consultation for validation and interpretation of findings.

**Results:**

Activity patterns differed by occupation. Farmers were more active through their work than other occupations, but were less active and more sedentary during the non-farming season than the farming season. Rural adults in Suixi generally had a low level of LTPA and a high level of TV viewing. Marital status, household size, social modelling for LTPA and owning sports equipment were significantly associated with LTPA but not with TV time. Most findings were validated through community consultation.

**Conclusions:**

For chronic disease prevention, attention should be paid to the currently decreasing occupational physical activity and increasing sedentary behaviours in rural China. Community and socially-based initiatives provide opportunities to promote LTPA and prevent further increase in sedentary behaviours.

## Introduction

Obesity is an emerging epidemic worldwide. In the last few decades, rapid urbanisation and economic growth in China have led to increasing prevalence of overweight and obesity [1]. China is in an epidemiological transition defined by positive energy balance and mounting prevalence of non-communicable diseases [2,3]. In 2000, 25.6% of the urban and 17.3% of the rural populations were overweight or obese, compared to 12.2% and 7.7% in 1989 [1].

In addition to a dramatic increase in energy intake [4,5], China has experienced a noticeable decrease in energy expenditure in both urban and rural areas [6]. Decreases in physical activity have been reported for several domains, including occupation, transportation and household activity [7]. In China, occupation has been a major source of physical activity [8]. According to the China Health and Nutrition Survey (CHNS), between 1991 and 2000, energy expenditure from occupational physical activity decreased by 22% among men and 24% among women, and the decrease contributed to the increase in body weight [9]. During the same nine-year period, energy expenditure from household physical activity decreased by 57% among men and 51% among women [9]. A similar trend was found with transportation physical activity [6]. As the Chinese society moves toward modern inactive lifestyles [7], leisure-time physical activity (LTPA) remains low [10]. Population survey data from 2006 found that only 13% of Chinese men and 8% of women engaged in any LTPA [6]. Although population data have shown a slight increase in LTPA, the increase has not been sufficient to compensate for declines in occupational and household activity [6].

Sedentary behaviour (too much sitting) is distinct from too little physical activity. Multiple studies have concluded that sedentary time is an independent risk factor for metabolic risk and chronic diseases [11,12]. TV viewing particularly has been associated with increased risks for overweight/obesity [13,14], diabetes [13,15], and metabolic syndrome [16,17]. Sedentary behaviours are understudied in China. One survey study in urban Qingdao found that adults spent an average of 3.2 sedentary hours per day watching TV, reading, or using a computer during non-working time [18]. Excess sedentary time is a public health concern in China given high rates of TV and personal computer ownership, particularly among urban residents [1]. No study could be found that examined sedentary behaviours in rural China.

In China, rural residents are currently more physically active and less obese than their urban counterparts [19]. However, a national study found a greater decrease in physical activity in rural areas than in urban areas over a recent ten-year period [20]. The current study was conducted in Suixi County, Guangdong, China, an area that has experienced farming-to-business shifts and some degree of economic modernisation, in common with many rural areas in China. Study objectives are: to assess levels of physical activity and sedentary behaviours of farming and non-farming adults in rural Suixi; to describe different activity patterns during farming and non-farming seasons; and to examine factors associated with LTPA and TV viewing.

## Methods

### Population and sampling

Participants were rural adults in Suixi County, Guangdong, China. The Suixi County population has been documented to be some 1.03 million (47% female), rural (>90% rural residents), and 77% of the working population are employed in farming or farming-related business/industry [21]. Households were randomly selected based on village, street, block, and house numbers. A list of 70 villages was enumerated based on information from the local government website http://cwgk.zhanjiang.gov.cn/. Ten villages were selected randomly. In each selected village, a map was obtained through local sources, and streets and blocks were randomly selected accordingly. In each selected household, the adult with the most recent birthday was invited to participate. If no one was home during the first visit, interviewers would return on the next two consecutive days for interview attempts. Inclusion criteria were 18 years or older and having lived in the village for at least three years. Written informed consent was obtained and small monetary incentives were provided. The San Diego State University Institutional Review Board approved the study.

In July 2009, 454 local residents were contacted and 287 participated in the survey (63% participation rate) during 14 days of data collection, which took place on both week days and weekend days depending on participants' availability. Interviewers kept notes of the gender and estimated age (young, middle-age, old) of each individual they approached and found no differential refusal rates by gender or age. Participants ranged from 18 to 82 years of age (mean = 40, SD = 16), 53% were women, 73% were married, and 65% completed middle school (nine-year mandatory education in China). About 32% were farmers, 18% were employed in non-farming occupations, 23% were self-employed shop keepers, and 26% were unemployed. The unemployed category included those who were retired, those not actively seeking employment, and those between jobs; the unemployed were usually not financially independent and were taken care of by families. It was possible, however, that they worked episodically for money.

### Procedures

Face-to-face interviews were conducted by trained interviewers, who were local college students speaking Mandarin and at least one of the two local dialects (Cantonese, Leizhou Dialect). Interviews were conducted in participant's preferred language. About 62% of interviews were conducted in Cantonese, 30% in Leizhou Dialect, and 8% in Mandarin. Verbal explanation and visual aids were provided if participants had problems understanding certain questions. For quality-control purposes, two interviewers were paired for each interview: one asked questions and wrote down answers while the other checked progress with the interview protocol and the accuracy of recorded answers.

### Questionnaire design

The questionnaire was developed from existing measures, informant interviews and focus groups. An iterative process of questionnaire development, informant interviews/focus groups, and questionnaire revision was used. Chinese-translated items were back-translated to English to compare with original questions. The final questionnaire was shortened, simplified, and was considered culturally appropriate by focus group participants, informants and interviewers.

### Measurement

#### Physical activity

Physical activity was measured in three domains: occupation, household, and leisure-time, using questions modelled after the Global Physical Activity Questionnaire (GPAQ) [22]. Occupational moderate-to-vigorous physical activity was defined as "activity that causes at least small increases in breathing and heart rate" through occupation; examples were provided such as lifting loads, digging, or farm work. Household physical activity was defined as any housework that involves physical activity, such as cleaning and maintaining the yard. Leisure-time physical activity (LTPA) was defined as any physical activity for the purpose of recreation and/or fitness, such as leisure walking, playing basketball, and martial arts. For each type of activity, two questions were asked about the number of days and hours/minutes per day in a *typical *week. A weekly time for each activity was calculated from the two questions.

#### Sedentary behaviour

This was defined as "sitting or reclining". Questions were modelled after GPAQ [22] regarding the days per *typical *week and time per day spent on each of the sedentary behaviours. Specific behaviours listed were suggested by focus groups as being "the most common sedentary activities" in rural Zhanjiang. These included TV viewing (when this was the primary behaviour and did not include doing other non-sedentary activities while TV was switched on [23]), recreational computer use (prevalent among young people), sitting chatting, playing Mahjong (a popular board game), and driving/riding a car/bus.

#### Seasonality of activity

Activity patterns of farmers are seasonal. Farmers do moderate-to-high-intensity, long-hour planting and harvesting activities during the farming season and less-intense field maintenance during the non-farming season. In the study sample, the length of the farming season ranged from two weeks to six months, depending on the type of crops under cultivation. To capture seasonality of activities, those in the farmer subsample was asked to estimate the length of farming season every year, and to recall physical activity and sedentary behaviours separately for a typical week during the farming and non-farming seasons.

#### Neighbourhood characteristics

The Neighborhood Environment Walkability Scale (NEWS) [24] aesthetics and safety subscales were translated and tested in formative interviews and focus groups. Based on their feedback, several items were deleted due to lack of relevance or appropriateness in rural China (e.g. crosswalks, posted speed limits). Items were simplified in language and response categories were reduced from four to two ("agree" vs. "disagree"). The final safety subscale included six items concerning walking/biking safety, crime rates, day-time safety, night-time safety, and street lights. The aesthetics subscale included three items regarding sidewalk condition, trees/shades, and cleanness.

#### Social modelling

Participants were asked "how many of your family members participate in physical activity for recreation or exercise?" Parallel questions were asked about friends and neighbours. Response options ranged from "none" (1) to "all" (5). Due to a large proportion of the "none" response, variables were dichotomized into "none" and "any".

### Data analyses

Activity data were examined for distribution and outliers. Outliers were identified and recoded to the 95th percentile of the distribution. Most of the physical activity and sedentary behaviour variables were highly skewed, and were therefore log transformed.

Analysis of covariance (ANCOVA) compared hours of physical activity and sedentary behaviours across occupations, adjusting for age as a covariate, and separately for men and women. Additional ANCOVA compared hours of activity between men and women for each employment category. For farmers, weighted physical activity hours were calculated based on the lengths of farming/non-farming seasons and activities in each season.

Participation in LTPA was dichotomized as "none" and "any" due to a large proportion of zero values (66%) and the lack of variance in LTPA time for those who reported any (around 80% reported an hour or less per week). TV time was median-split at 12 hours/week. χ^2 ^tests and t-tests examined bivariate associations between independent variables and dependent variables (LTPA, TV time).Variables with a significance level of p < 0.05 were included in the multiple logistic regression model. Analyses were first conducted for men and women separately, and then combined if similar associations were observed for both genders. Social-modelling variables were summed as an overall index in the logistic regression model.

### Informant consultation

Member checking [25], a qualitative research method, was used to obtain community feedback and to validate findings. Informants (n = 10) were rural adults who did not participate in the previous survey, and were randomly selected from the villages where the survey interviews were conducted. Key findings from quantitative data analyses were abstracted into simple sentences and graphs, and were presented to informants both individually and as a group. Informants were encouraged to provide feedback and interpretation of findings, which were audio taped and transcribed.

## Results

### Descriptive statistics of physical activity and sedentary behaviours

As Table 1 shows, farmers had more occupational physical activity compared to other groups. Farmers and those who were self-employed had more TV viewing time than did non-farming, and unemployed respondents. Farmers reported almost no recreational computer use, and more sitting chatting time compared to other occupations (statistically significant only among women).

**Table 1:** Physical activity (PA) and sedentary behaviours by occupation and gender in rural Suixi, Guangdong, China^a^

	**Farming**^**b**^	Non-farming Employed	Self-employed (Shop keeping)	Unemployed	*p*
**Men**	n = 46	n = 34	n = 35	n = 37	
Occupational PA	9.98	2.13	1.50	NA	**<0.001**
Household PA	4.87**	3.22*	3.85*	3.50**	0.772
Leisure-time PA	0.35	0.93	0.68	1.27	0.141
Sedentary Behaviours					
Recreational computer use	0.01	0.93	0.86	1.56	**<0.001**
Playing majiang	0.86	0.72	0.67	1.46	0.260
Watching TV	13.57	8.76	14.20	9.49	**0.045**
Driving/riding in a car	1.15	1.80*	1.77*	1.41	0.349
Sitting chatting	4.03	2.19	1.59	2.90	0.130

**Women**	n = 45	n = 19	n = 32	n = 39	
Occupational PA	9.65	1.48	1.72	NA	**<0.001**
Household PA	13.08**	11.55*	11.06*	11.30**	0.478
Leisure-time PA	0.23	0.72	0.25	0.32	0.116
Sedentary Behaviours					
Recreational computer use	0.00	0.38	0.40	1.02	**<0.001**
Playing majiang	0.51	0.55	0.49	0.42	0.659
Watching TV	13.80	7.39	12.68	7.50	**0.047**
Driving/riding in a car	0.66	0.49*	0.55*	0.32	0.536
Sitting chatting	3.75	1.25	2.03	1.66	**0.014**

^a ^Age-adjusted geometric mean based on ANCOVA (unit: hour/week)

^b ^Activity data for farmers were weighted on length of farming/non-farming seasons

**p < 0.01, *p < 0.05 for comparison between men and women

In general, men and women showed similar activity patterns across occupation categories. Geometric means for different types of activity were similar between genders. Exceptions were that women spent much more time on household physical activity and less time on motorized transportation.

### Seasonal activity patterns for farmers

**Figure 1:**
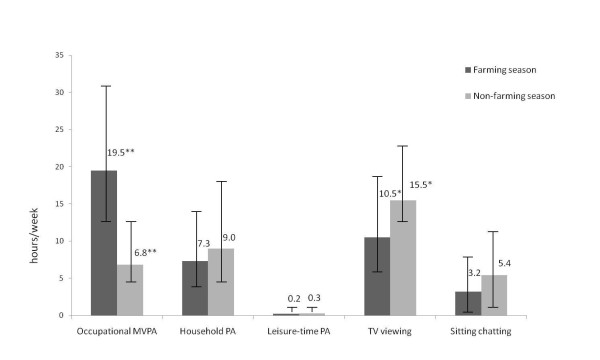
Hours per week (geometric mean, 95% confidence interval) of physical activity and sedentary behaviours of farmers during the farming and non-farming seasons (* p < 0.05, **P < 0.01).

Compared to the farming season, farmers had significantly less occupational physical activity (geometric mean 6.79 vs. 19.54 hr/week, p < 0.01) and more TV viewing (15.54 vs. 10.45, p < 0.05) during the non-farming season (Figure 1). LTPA level was low for both the farming and non-farming seasons (0.22 vs. 0.33, p = 0.340).

### Correlates of leisure-time physical activity and TV time

Bivariate analyses (Table 2) showed higher participation in LTPA among those who were younger, men, single, middle school graduated, living in a smaller household, employed in non-farming occupations or not employed, having sports equipment in the home, living in a safer neighbourhood, and having family, friends, and neighbours who exercised regularly.

**Table 2:** Leisure-time physical activity (LTPA) and weekly TV viewing time by demographic, individual, environmental and social variables in rural Suixi, Guangdong, China

	Any LTPA (n = 99)	No LTPA (n = 188)	***p***^**a**^	TV < 12 hr/week (n = 148)	TV > = 12 hr/week (n = 139)	***p***^**a**^
**Demographic attributes**						
Age (year, mean ± SD)	31.7 ± 14.2	44.1 ± 15.6	**<0.001**	39.6 ± 15.8	40.4 ± 15.8	0.691
Women (%)	36.4	52.7	**0.006**	54.1	51.8	0.724
Married (%)	49.5	85.1	**<0.001**	72.3	73.4	0.895
Middle school graduate (%)	85.9	53.2	**<0.001**	67.6	61.2	0.269
Occupation (%)			**<0.001**			0.230
Farming	17.3	44.5		33.8	36.2	
Non-farming	30.6	11.5		20.4	15.9	
Self-employed (shop keeping)	22.4	25.8		20.4	29.0	
Unemployed	29.6	18.1		25.4	18.8	
Household size (mean ± SD)	5.0 ± 1.8	6.0 ± 2.8	**<0.001**	5.9 ± 2.5	5.5 ± 2.6	0.840
**Individual behaviour**						
Current smoking (%)	30.9	33.5	0.690	32.2	33.1	0.790
Current drinking (%)	35.4	30.3	0.425	35.1	28.8	0.235
Weekly TV time (hour, mean ± SD)	13.0 ± 8.1	12.4 ± 7.6	0.495	----	----	----
**Environmental variables**						
Having sports equipment in the home (%)	68.4	40.3	**<0.001**	46.4	54.0	0.209
Neighbourhood crime-related safety^b ^(mean ± SD)	4.5 ± 1.1	4.1 ± 1.2	**0.017**	4.3 ± 1.2	4.2 ± 1.1	0.743
Neighbourhood aesthetic^c ^(mean ± SD)	1.8 ± 1.0	1.8 ± 1.0	0.554	1.7 ± 1.0	1.9 ± 1.0	0.070
**Social modelling**						
Family exercise regularly (%)	86.9	26.6	**<0.001**	45.9	48.9	0.614
Friends exercise regularly (%)	69.7	26.6	**<0.001**	38.5	44.6	0.295
Neighbours exercise regularly (%)	67.7	26.1	**<0.001**	40.5	40.3	0.965

^a ^Based on t-test for continuous variables and *χ*^*2*^testsfor categorical variables

^b ^Neighbourhood crime-related safety score ranges 0 to 6; a higher score indicates better safety

^c ^Neighbourhood aesthetic score ranges 0 to 3; a higher score indicates better aesthetic/maintenance conditions

In the multiple logistic regression model (Table 3), those who were married had less than one third the odds of participating in LTPA compared to those who were single. An additional person living in the household was associated with a 21% decrease in odds of LTPA. Those who had sports equipment in the home had twice the odds of participating in LTPA. A one-unit increase in the social modelling scale was associated with a more than two-fold increase in the odds of LTPA. Similar patterns of associations were found among men and women.

**Table 3:** Multiple logistic regression analysis of leisure-time physical activity (LTPA)^a ^in rural Suixi, Guangdong, China (n = 287)

Variables	OR	95% CI	p
Age (continuous)	0.98	0.95, 1.01	0.197
			
Gender			
Women	1.00		
Men	1.57	0.77, 3.18	0.214
			
Marital status			
Single/divorced/widowed	1.00		
Married	0.27	0.11, 0.07	**0.007**
			
Number of residents in the household (continuous)	0.79	0.68, 0.92	**0.003**
			
Education			
Lower than middle school	1.00		
Middle school graduate or higher	2.44	0.96, 6.16	0.060
			
Occupation			
Farming	1.00		
Non-farming	2.37	0.84,6.70	
Self-employed	0.46	0.16,1.28	
Unemployed	0.49	0.12,1.30	**0.008**
			
Neighbourhood crime-related safety^b^	1.20	0.90, 1.62	0.163
			
Sports equipment in the home			
No	1.00		
Yes	2.03	1.03, 4.05	**0.039**
			
Social modelling of leisure-time physical activity^c^	2.36	1.76, 3.16	**<0.001**

^a ^LTPA is coded 1 = any, 0 = none

^b ^Neighbourhood crime-related safety score ranges 0 to 6; a higher score indicates better safety

^c ^Social modelling score ranges 0 to 3; a higher score indicates more family members, friends, and neighbours participating in leisure-time physical activity

Gender-stratified and combined bivariate analyses and multiple logistic regressions suggested that none of the correlates tested was significantly associated with TV viewing time; therefore, no logistic regression model is presented.

## Discussion

This study in rural Suixi found differences in physical activity and sedentary behaviour by occupation. Farmers had significantly more occupational physical activity compared to their non-farming counterparts, however, they appeared to substitute farming activities with sedentary behaviours (primarily TV viewing) during non-farming seasons. Based on this pattern, one may expect further decreases in occupational physical activity as a result of fewer farming-related occupations, less labour-intensive farm work, and shorter farming seasons. Without compensating forms of physical activity or reduction of TV time, this pattern suggests future increases in overweight/obesity and associated chronic diseases.

The prevalence of LTPA was low in the study sample, which was consistent with findings from previous studies [6,10,19]. Correlates of LTPA were explored. After adjusting for other demographic characteristics and environmental variables, those in our study were more likely to participate in LTPA if they were unmarried, living in a smaller household, having sports equipment in the home and if their family, friends, or neighbours participated in LTPA.

The strongest correlate of LTPA was social modelling. During the "member checking" phase of the study, informants strongly agreed with this finding. A woman informant stated, "We are in a very collective society and nobody exercises alone." Other informants confirmed that families, friends, and neighbours in their communities were closely connected (i.e., "The social circle is small here. If one person goes out for a walk, immediately others will notice and probably will follow. They can chat while walking.") Studies in western countries have suggested that seeing other people exercise was associated with individuals' own participation in LTPA, possibly through modelling and prompting [26-28]. In the context of rural China, exercising with others may provide additional and possibly essential social reinforcement. Social support and group activity interventions are effective in Western countries [29], and they may be even more effective in China, but this remains to be demonstrated.

Another finding was that those who had sports equipment in the home had twice the odds of engaging in LTPA. In a cross-sectional study, such a finding should be interpreted with caution, since it is possible that those who had already been active would be more likely to obtain such equipment. Informants suggested that home equipment was only half of the equation if access to exercise facilities and localities was limited. For example, a young man stated "It is true that I am more active because I have a basketball, but I play in streets where it is not safe. I would have played a lot more if there was a basketball court around." Although the Chinese government has implemented the "Sports for All" program to expand relevant facilities and infrastructure [30], rural residents still have limited access to such facilities, as demonstrated by one informant's comment, "Sometimes we have to travel for hours to a bigger town for sports. We do not have anything here in our village." In this study sample, adults who were married or who were living in a larger household were less active. This may be a result of related lifestyles and competing priorities of other household activities. An informant said: "We have to travel so far for any activity. If one is married and has children (like me), how can s/he have time for any recreation?"

TV viewing was the major sedentary behaviour. The median TV time was 12 hours per week, and 39% watched TV for 14 hours or more. This is comparable to TV viewing time reported by adults in the US [31,32]. A woman informant described the "common" lifestyle in her village: "People in my village tend to turn on TV immediately after meals, and then they rest (sitting or lying down) for one hour after lunch, and another hour after dinner." In the current sample, 96% of participants had a TV set in the home, indicating substantial influences from modernisation. Although computers were not as common (23% participants had a computer at home), it is reasonable to postulate that as computer ownership increases, screen time sedentary behaviour may further increase in rural China.

None of the correlates of LTPA was significantly associated with TV time. This finding was similar to a previous study, in which environmental variables that facilitated physical activity were not related to TV time [33]. Furthermore, no association was found between TV time and LTPA in the current sample (Table 2). This suggests that TV viewing may be a behaviour that is independent of physical activity in rural Suixi [11].

A major strength of the study was using community participation to enhance the design, survey administration and results interpretation. These procedures improved the cultural relevance of research questions, qualitatively validated research findings, and provided interpretations that extended quantitative results. Although a small sample from one county in southern China limits generalizability, and a cross-sectional study cannot establish causality, these findings provide insights to guide future larger-scale and prospective studies that employ objective measures of the environment, physical activity, multiple sedentary behaviours (including sleeping) [34], and health outcomes. Future studies should assess additional aspects of the built and social environments and explore variables related to sedentary behaviours, especially TV viewing.

## Conclusions

The current study, together with previous studies on the consequences of modernisation in China [6,9,10,35], suggests that if no effective public health actions or social and environmental initiatives are implemented, further decreases in physical activity and increases in sedentary behaviours leading to higher risk of major chronic diseases are likely, as a by-product of the economic development, modernisation, and urbanisation in China.

In rapid social and epidemiological transitions, Chinese central and local governments are in the position of making health-promoting changes and taking preventive measures through city planning initiatives, infrastructure building, and land-use development to provide activity environments [36]. Physical activity programs should be implemented in rural China targeting multiple levels of influences [37]. Interventions to improve accessibility of recreational facilities and to promote physical activity as a social behaviour may be culturally tailored.

## Competing interests

The authors declare that they have no competing interests.

## Authors' contributions

DD designed the study, developed research plans and instrument, conducted focus group interviews, data collection, analyses, community consultation, and drafted the manuscript. MFH and JFS provided guidance and advice throughout the entire study and contributed to the writing of the manuscript. JD coordinated the involvement of Zhanjiang Normal University, provided input to study design, organized trainings, and supervised interviewers. MZ and HH organized focus group interviews, provided input to instrument development, and conducted interviews. NO provided guidance on the focus and organisation of content, and contributed to the writing of the manuscript. All authors have read and approved the final manuscript.
